# Radiation-associated cerebroophtalmic effects

**DOI:** 10.1192/j.eurpsy.2022.738

**Published:** 2022-09-01

**Authors:** K. Loganovsky, P. Fedirko, D. Marazziti, T. Loganovskaja, K. Kuts, I. Perchuk, T. Babenko, K. Antypchuk, G. Kreinis

**Affiliations:** 1 State Institution “National Research Centre for Radiation Medicine of National Academy of Medical Sciences of Ukraine”, Radiation Psychoneurology, Institute Of Clinical Radiology, Kyiv, Ukraine; 2 State Institution “National Research Centre for Radiation Medicine of National Academy of Medical Sciences of Ukraine”, Institute Of Radiation Hygiene And Epidemiology, Kyiv, Ukraine; 3 University of Pisa, Clinical And Experimental Medicine, Section Of Psychiatry, Pisa, Italy

**Keywords:** Chernobyl accident, comorbidity, Ionizing radiation, cerebroophtalmic effects

## Abstract

**Introduction:**

We proposed to consider the brain and eye as a target of ionizing radiation exposure. Prevention of potential radiation-associated cerebroophtalmic effects are crucial for successful long-term space missions; interventional radiology; medical, occupational and accidental irradiation

**Objectives:**

Determination of radiation-associated cerebroophtalmic effects in the long term after irradiation in adulthood and *in utero.*

**Methods:**

Neuropsychiatric, ophtalmological, neurophysiological and neuropsychological assessment of irradiated in adulthood (57 Chernobyl accident clean-up workers, liquidators), 52 persons exposed *in utero* as a result of the Chernobyl accident, comparison group (51 combatants of the Antiterrorist operation in Donbass), and 53 healthy people.

**Results:**

Radiation-associated cerebroophthalmic pathology is characterized by high neuropsychiatric and ophthalmic comorbidity, which increases in proportion to the radiation dose, and is mainly represented by chronic vascular and degenerative diseases of the brain and retina, mild cognitive impairment (after irradiation in adulthood), as well as disorders of the autonomic nervous system; non-psychotic organic mental disorders; neurotic, stress-related and somatoform disorders; vascular and dystrophic processes in the retina (after *in utero* exposure). Characteristic of both radiological scenarios remains intellectual disharmony due to a decrease in the verbal IQ. The delay and attenuation of cerebral visual afferentation processing were observed in prenatally exposed.

**Conclusions:**

Radiation-associated cerebroophtalmic effects in the long term after irradiation in adulthood and *in utero* could be mainly classified as a “small vessel disease of the brain and eye” of vascular-degenerative nature and possible latent demyelination after irradiation *in utero.*

**Disclosure:**

No significant relationships.

